# Behavioral Clusters of Diet, Physical Activity, and Sedentary Behavior Among Moroccan School-Age Adolescents: A Latent Class Analysis

**DOI:** 10.7759/cureus.96125

**Published:** 2025-11-05

**Authors:** Siham Bouftini, Youness El Achhab, Hicham El Kazdouh, Abdelfettah El-Ammari, Abdelghaffar El-Ammari

**Affiliations:** 1 Epidemiology and Public Health, Higher Institute of Nursing Professions and Health Techniques (ISPITS) Ministry of Health and Social Protection, Rabat, MAR; 2 Epidemiology and Public Health, Regional Center for Education and Training Professions (CRMEF), Fez, MAR; 3 Epidemiology and Public Health, Euro-Mediterranean University of Fez (UEMF), Fez, MAR; 4 Biological Sciences, Regional Center for Education and Training Professions (CRMEF), Tangier, MAR; 5 Epidemiology and Public Health, Laboratory of Epidemiology and Research in Health Sciences, Faculty of Medicine, Pharmacy and Dental Medicine (FMPMD) Sidi Mohamed Ben Abdellah University (USMBA), Fez, MAR; 6 Epidemiology and Public Health, National School of Public Health (ENSP) Ministry of Health and Social Protection, Rabat, MAR

**Keywords:** adolescents, clustering, latent class analysis, mental health, morocco, physical activity, sedentary behavior, smoking, unhealthy diet

## Abstract

Introduction: Adolescence is a critical period when unhealthy diet (UD), insufficient physical activity (PA), and sedentary behavior (SB) co-occur and persist into adulthood, fueling noncommunicable diseases. This is the first national latent class analysis (LCA) of UD, PA, and SB among Moroccan adolescents. The objective of this study was to identify latent behavioral classes and to examine their sociodemographic and mental health correlates.

Methods: We analyzed cross-sectional data from the 2016 Global School-based Student Health Survey (GSHS) in Morocco. Unweighted LCA was applied to binary indicators of UD, insufficient PA, and SB. Models with two to five classes were estimated, and we retained the most parsimonious and substantively interpretable solution. Correlates of class membership were estimated with multinomial logistic regression.

Results: Complete data were available for 6,745 school-attending adolescents (median age 15 years; 46.9% female; 48.8% urban; response rate 91%). A three-class solution provided the best fit: “low fruit/vegetable intake and inactive” (58.1%), “carbonated soft drink and fast-food intake and inactive” (18.8%), and “multi-risk diet and inactive” (23.1%). Compared with the “low fruit/vegetable intake and inactive” class, higher odds of membership in the two higher risk profiles were observed for urban residence, male sex (for the multi-risk diet and inactive class), high-school level, anxiety, suicidality, and smoking; underweight status was associated with lower odds for the multi-risk profile.

Conclusions: To our knowledge, this is the first national LCA of UD, insufficient PA, and SB among Moroccan adolescents, identifying three recurrent profiles all underpinned by insufficient PA and differentiated by diet. Findings inform policy and practice, supporting school-based, multi-behavior programs pairing PA promotion with dietary improvements and brief mental health support, with priority for boys, urban schools, and smokers. Estimates are sample-specific; future work should use survey-weighted/three-step LCA and longitudinal, multilevel designs incorporating sleep and objective PA.

## Introduction

Although adolescence is often considered a healthy life stage, multiple risk behaviors, including unhealthy diet (UD), insufficient physical activity (PA), and sedentary behavior (SB), emerge and compromise current and future health [1]. Despite known harms, many adolescents do not meet guidelines for diet and PA [2-5]. In Morocco’s Global School-based Student Health Survey (GSHS), 67.7% reported inadequate fruit and vegetable (FV) intake (< 5 servings/day), 89.2% did not achieve ≥ 60 minutes/day of PA, and about 30% accumulated ≥ 3 hours/day in sitting activities [6]. Interventions are needed, as behaviors adopted in adolescence often track into adulthood [1].

UD, insufficient PA, and SB rarely occur in isolation but cluster into lifestyle patterns that are jointly associated with poorer physical and mental health and may have synergistic effects beyond single behaviors [7]. Beyond cross-sectional co-occurrence, a recent systematic review of 18 prospective studies (n = 26,772) showed strong tracking of clusters from childhood through adolescence (64% with stable transition probabilities of 60-100%), and remaining in unhealthy clusters was associated with higher adiposity [8].

Across adolescent samples, three broad lifestyle profiles are consistently observed (healthy, unhealthy, and mixed), with the mixed profile being the most common [7,9-11]. In a longitudinal cohort (n = 3,065), three clusters were identified at age 14; membership in the “healthy” cluster was associated with lower body mass index (BMI) z-score and percent body fat at 14 that remained lower at 17 [11]. Behavioral clusters show clear sociodemographic patterning: girls more often occupy high-diet-quality/low-PA/high-SB profiles, boys mixed high PA/high SB/poorer diet profiles; unhealthy profiles concentrate in lower socioeconomic status (SES) groups and are linked to poorer physical and mental health, whereas healthier profiles relate to better social well-being [7,12].

Rather than treating behaviors in isolation, the clustering approach examines how they co-occur within individuals and can guide multi-behavior interventions [13]. Evidence from low- and middle-income countries (LMICs) remains scarce, including in Morocco, where economic, sociocultural, and demographic factors likely shape clustering [14,15]. Patterns also differ across subpopulations, yet adolescent data from LMICs are limited [9]. Analyses using LCA at the national level are scarce in LMICs, particularly in Morocco.

To help address this gap, this study had two main aims: (i) to identify distinct behavioral classes of UD, insufficient PA, and SB among school-age adolescents in Morocco, using LCA and (ii) to examine the sociodemographic and mental health factors associated with these classes.

## Materials and methods

Data source

We analyzed data from the nationally representative 2016 Morocco GSHS [6], a school-based, cross-sectional survey of in-school adolescents aged 13-17 years, developed by the World Health Organization (WHO) and the United States Centers for Disease Control and Prevention (CDC). Anonymous, standardized questionnaires were administered during class. A two-stage cluster sampling was used: schools were selected with probability proportional to enrollment, then random sampling of classes with all students invited to participate [6].

Variable measurement

Unless otherwise noted, variables were recoded as binary (1 = higher-risk category; 0 = reference), and missing responses were set to missing and excluded from analysis. All items were taken from the standardized WHO/CDC GSHS core questionnaire for Morocco 2016 [6]. Specifically, insufficient PA was assessed with: “During the past seven days, on how many days were you physically active for a total of at least 60 minutes per day? Add up all the time you spent in any kind of physical activity each day,” using an eight-point scale (1 = 0 days, …, 8 = 7 days), and was recoded as inactive = 0-6 days (1) versus active = 7/7 days (0), consistent with the ≥ 60 min/day guideline.

Similarly, SB was assessed with: “On a typical day, how much time do you spend sitting and watching television, playing computer games, talking with friends, or doing other sitting activities such as using the computer or cell phone?” on a six-point scale (1 = 0 h/day, …, 6 = ≥ 8 h/day), and was recoded as sedentary = ≥ 3 h/day (1) versus < 3 h/day (0). In parallel, a UD was operationalized with three indicators. First, FV intake was measured with two items (seven-point scales, 1 = none to 7 = ≥ 5 times/day) and combined to classify inadequate intake as < 5 total servings/day (1) versus adequate intake ≥ 5 servings/day (0). Second, carbonated soft drink (CSD) intake was measured with one item on usual times per day in the past 30 days (seven-point scale, 1 = none to 7 = ≥ 5 times/day) and recoded as CSD ≥ 1 time/day (1) versus < 1 time/day (0). Third, fast-food (FF) consumption was assessed with one item on the number of days in the past seven days the respondent ate food from a fast-food restaurant (eight-point scale, 1 = 0 days to 8 = 7 days) and recoded as ≥ 1 day (1) versus 0 days (0).

Finally, covariates included area of residence (urban/rural), sex, age, grade/academic level, SES, BMI, loneliness, anxiety (single-item self-reports), suicidal ideation, suicidal plan, suicidal attempt (assessed as separate single items), and current cigarette smoking. Detailed definitions and coding of covariates (including SES) are provided in the Appendices. Variables were dichotomized to align with established public health cutoffs and guidelines (e.g., WHO/CDC recommendations for PA and SB [2,3]) and to follow the official GSHS Data User’s Guide and Public Use Codebook [6]. This ensures comparability with GSHS analyses, improves interpretability and model stability in the LCA, and promotes parsimony by reducing the number of parameters to estimate [13,15].

Data analysis

We first ran descriptive analyses to summarize sociodemographic characteristics and health behaviors. We then fitted LCA models to identify unobserved subgroups defined by response patterns on the binary indicators of UD, PA, and SB (person-centered approach). We sequentially estimated two- to five-class solutions and retained the most parsimonious, substantively interpretable model [13,15]. We compared solutions using model fit indices, including the Akaike information criterion (AIC), Bayesian information criterion (BIC), and Bozdogan’s consistent AIC (CAIC). Lower values for these information criteria indicate a better balance between model fit and parsimony.

We also evaluated entropy (0-1), with higher values indicating clearer class separation. We reported the likelihood-ratio goodness-of-fit statistic G² (as χ² with p-value) and used the bootstrapped likelihood-ratio test (BLRT) to compare K vs K−1 solutions (with K denoting the number of classes), with test statistic 2ΔLL = 2 × (LL_K − LL_(K−1)) (with LL = log-likelihood) and parametric-bootstrap p-values; a significant BLRT favors the more complex model [13].

To assess the local independence assumption, we inspected bivariate residuals (BVR) for each pair of indicators under the selected model; for 2 × 2 tables, BVR > 3.84 (χ² with df = 1, α=0.05) was treated as evidence of local dependence [16]. Models were estimated via the expectation-maximization (EM) algorithm with multiple random starts, and we retained the solution with the maximum log-likelihood [13,15]. Individuals were assigned to their most likely class (modal assignment) [13]. Finally, associations between class membership and covariates were estimated using multinomial logistic regression with Class 1 as the reference; all covariates were entered simultaneously (mutually adjusted). The study was not preregistered; analyses were primarily exploratory. Data management was performed in Microsoft Excel (Microsoft Corporation, Redmond, Washington, United States), and statistical analyses were conducted in Jamovi (The jamovi project; https://www.jamovi.org) and R (R Foundation for Statistical Computing, Vienna, Austria, https://www.R-project.org/; https://github.com/tidyverse/ggplot2).

## Results

Descriptive results

Complete data were available for 6,745 participants. The median age was 15 years; 48.8% lived in urban areas, and 46.9% were female. Regarding behaviors, 67.7% reported inadequate FV intake, 32.5% consumed CSD, 62.8% consumed FF, 89.2% did not achieve ≥ 60 minutes/day of PA, and 29.2% accumulated ≥ 3 hours/day in sitting activities. The characteristics are shown in Table 1.

**Table 1:** Overview of sociodemographic characteristics and selected health behaviors in a national sample of Moroccan school-age adolescents (N = 6,745), GSHS 2016. GSHS: Global School-based Student Health Survey [6]

Characteristics	Frequency (Percentage)
Sociodemographic characteristics	
Area	
Rural	3452 (51.2)
Urban	3293 (48.8)
Sex	
Girl	3085 (46.9)
Boy	3488 (53.1)
Age (years)	
13 or less	1863 (28.1)
14-15	2212 (33.3)
16 or more	2558 (38.6)
Academic level (grades)	
Middle school (7^th^, 8^th^, and 9^th^)	4381 (66.9)
High school (10^th^, 11^th^, and 12^th^)	2164 (33.1)
Socioeconomic status (SES)	
Low	680 (10.4)
Medium or High	5859 (89.6)
Body mass index (BMI)	
Normal	4761 (78.7)
Underweight	507 (8.4)
Overweight or obese	780 (12.9)
Unhealthy dietary behaviors and physical inactivity	
Inadequate fruit and vegetable intake	4566 (67.7)
Carbonated soft drink consumption	2162 (32.5)
Fast food consumption	4196 (62.8)
Physical inactivity	5883 (89.2)
Sedentary behavior	1891 (29.2)
Poor mental health	
Loneliness	1317 (20.1)
Anxiety	1171 (17.6)
Suicidal ideation	1031 (16.0)
Suicide plans	932 (14.6)
Suicide attempts	939 (14.2)
Suicidal ideation, plan, or attempt	1513 (23.1)
Cigarette smoking	534 (8.3)

Latent class models (model fit and class selection)

We compared two- to five-class solutions (Table 2). The three-class solution had the lowest BIC (with competitive AIC/CAIC), acceptable entropy (0.59, indicating moderate class separation), and no class < 10% (smallest class 18.8%). The BLRT favored K over K−1 (p = 0.04). All BVRs were < 3.84, supporting local independence (maximum BVR = 1.04 for inadequate FV × insufficient PA). We therefore retained the three-class model for interpretation.

**Table 2:** Fit Indices for two- to five-class latent class solutions on UD (Inadequate FV, CSD, FF), Insufficient PA, and SB in the 2016 Moroccan GSHS Analytic Sample (N = 6,745). 2ΔLL: 2 × (LL_k − LL_(k−1)). A dash (—) indicates not applicable because the BLRT statistic for the 2-class solution would compare against the 1-class model, which is not shown here. Lower is better for AIC, CAIC (Consistent AIC), BIC, and G²; higher is better for entropy. LL: log-likelihood; AIC: Akaike Information Criterion; CAIC: consistent Akaike Information Criterion; BIC: Bayesian Information Criterion; G²: maximum likelihood-ratio test; BLRT: bootstrapped likelihood-ratio test; FV: fruit and vegetable intake; FF: fast food; CSD: carbonated soft drink; UD: unhealthy diet; PA: physical activity; SB: sedentary behavior; GSHS: Global School-based Student Health Survey [6]

Number of classes	Model fit indices							
	Log-likelihood	AIC	CAIC	BIC	Entropy	G²	BLRT (2ΔLL)	BLRT (p-value)
2	-18674	37369	37455	37444	0.534	109.70	—	< .001
3	-18630	37293	37426	37409	0.594	21.85	88.00	0.040
4	-18624	37293	37473	37450	0.638	9.79	12.00	0.200
5	-18623	37304	37531	37502	0.590	8.55	2.00	0.080

Latent class and item-response probabilities

**Figure 1:**
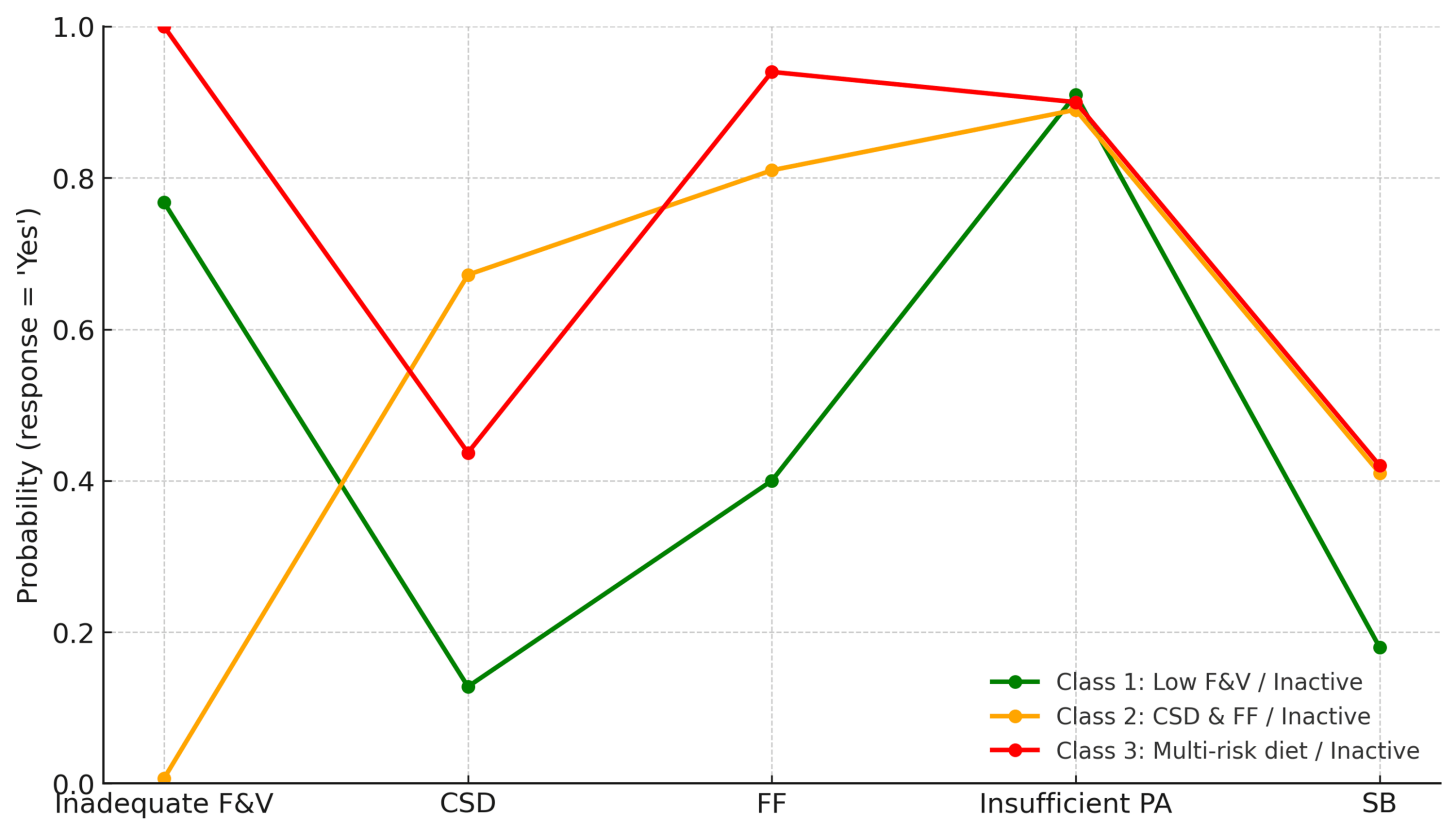
Profile plot for the three-class LCA on unhealthy diets (FV, CSD, FF), insufficient PA, and SB in the 2016 Moroccan GSHS analytic sample (N = 6,745). FV: fruit and vegetable intake; FF: fast food; CSD: carbonated soft drink; UD: unhealthy diet; PA: physical activity; SB: sedentary behavior; LCA: latent class analysis; GSHS: Global School-based Student Health Survey [6]

Class-specific conditional probabilities (Table 3) and the profile plot (Figure 1) indicate the following: First, Class 1 - “Low FV / Inactive” (58.1%): high probability of inadequate FV intake (0.77) and insufficient PA (0.91). Second, Class 2 - “CSD and FF/Inactive” (18.8%): high probability of CSD consumption (0.67), FF consumption (0.81), and insufficient PA (0.89). Finally, Class 3 - “Multi-risk diet/Inactive” (23.1%): very high probabilities of inadequate FV intake (0.99), FF consumption (0.94), and insufficient PA (0.90), with moderate probabilities of CSD consumption (0.44) and SB (0.42).

**Table 3:** Summary of latent-class profiles: class sizes and conditional probabilities. Notes. Class 1 (Low FV/Inactive); Class 2 (CSD and FF/Inactive); Class 3 (Multi-risk diet/Inactive)

Latent class indicators		Class 1	Class 2	Class 3
Class size (n, %)		3917 (58.1 %)	1270 (18.8 %)	1558 (23.1 %)
Inadequate fruit and vegetable (FV) intake	Yes	0.768	0.007	1.000
	No	0.232	0.993	0.000
Carbonated soft drink (CSD) consumption	Yes	0.128	0.672	0.437
	No	0.872	0.328	0.563
Fast food (FF) consumption	Yes	0.388	0.814	0.939
	No	0.612	0.1857	0.061
Insufficient physical activity (PA)	Yes	0.908	0.845	0.895
	No	0.092	0.1555	0.105
Sedentary behavior (SB)	Yes	0.188	0.387	0.421
	No	0.812	0.613	0.579

Factors associated with cluster membership

Compared with Class 1, adolescents in Class 2 had higher odds for urban residence (OR = 1.16; 95%CI = 1.01-1.34), suicidal ideation/plan/attempt (OR = 1.39; 95%CI = 1.16-1.65), and smoking (OR = 1.82; 95%CI = 1.38-2.42), while being in high school was associated with lower odds (OR = 0.63; 95%CI = 0.53-0.74). For Class 3 vs Class 1, higher odds were observed for urban residence (OR = 1.49; 95%CI = 1.30-1.70), male sex (OR = 1.23; 95%CI = 1.07-1.41), high-school level (OR = 1.26; 95%CI = 1.10-1.45), anxiety (OR = 1.26; 95%CI = 1.06-1.50), suicidal ideation/plan/attempt (OR = 1.46; 95%CI = 1.23-1.72), and smoking (OR = 2.32; 95%CI = 1.82-2.96), whereas underweight was inversely associated (OR = 0.70; 95%CI = 0.54-0.91) (Table 4).

**Table 4:** Multinomial logistic regression of correlates of latent class membership in the 2016 Moroccan GSHS analytic sample of school-attending adolescents (N = 6,745). Class 1: (Low FV/Inactive) is the Reference category; Class 2: (CSD and FF/Inactive); Class 3: (Multi-risk diet/Inactive); Wald χ² (df = 1) and p-values are from Wald tests of multinomial logistic regression coefficients. A dash (—) indicates “not applicable” for reference categories GSHS: Global School-based Student Health Survey; FF: fast food; CSD: carbonated soft drink

Covariates	Class 2 OR (95% CI)	Wald χ² (p)	Class 3 OR (95% CI)	Wald χ² (p)
Area of residence				
Rural (Ref)	—	—	—	—
Urban	1.16 (1.01-1.34)	4.23 (0.040)	1.49 (1.30-1.70)	33.96 (<0.001)
Sex				
Girl (Ref)	—	—	—	—
Boy	0.93 (0.80-1.07)	0.96 (0.328)	1.23 (1.07-1.41)	8.65 (0.003)
Academic level				
Middle school (Ref)	—	—	—	—
High school	0.63 (0.53-0.74)	29.45 (<0.001)	1.26 (1.10-1.45)	10.75 (0.001)
Body mass index (BMI)				
Normal weight (Ref)	—	—	—	—
Underweight	0.95 (0.74-1.23)	0.16 (0.692)	0.70 (0.54-0.91)	7.18 (0.007)
Overweight or obese	1.11 (0.90-1.36)	0.98 (0.322)	0.94 (0.77-1.15)	0.37 (0.545)
Anxiety				
No (Ref)	—	—	—	—
Yes	1.10 (0.91-1.33)	0.97 (0.325)	1.26 (1.06-1.50)	6.81 (0.009)
Suicidal ideation, plan or attempt				
No (Ref)	—	—	—	—
Yes	1.39 (1.16-1.65)	13.42 (<0.001)	1.46 (1.23-1.72)	19.57 (<0.001)
Cigarette smoking				
No (Ref)	—	—	—	—
Yes	1.82 (1.38-2.42)	17.47 (<0.001)	2.32 (1.82-2.96)	46.01 (<0.001)

## Discussion

This study used LCA to identify latent behavioral classes of UD, PA, and SB among Moroccan adolescents and to examine their sociodemographic and mental health correlates. A three-class solution fit best: low FV intake and inactive (58.1%), CSD and FF intake and inactive (18.8%), and multi-risk diet and inactive (23.1%). Urban residence, male sex (for the multi-risk class), high-school level, poor mental health indicators (anxiety and suicidal behaviors), and smoking were associated with higher odds of membership in the riskier classes, while underweight was linked to lower odds for the multi-risk class. Insufficient PA was common to all profiles, underscoring the magnitude of this issue among adolescents and the need for initiatives to reverse the trend [17]. No profile was completely healthy; each combined at least two unfavorable behaviors, consistent with prior work showing that UD, PA, and SB cluster in complex ways among youth, with mixed profiles frequently observed [9,10,14].

Briefly, theories such as Compensatory Health Beliefs Theory (CHBT) and Problem Behavior Theory (PBT) have been advanced to explain why healthy and unhealthy behaviors may co-occur within the same individuals [10]. According to CHBT, individuals may perceive a healthy behavior (e.g., FV intake) to compensate for an unhealthy one (e.g., CSD). This theory suggests a balanced approach, where positive actions are used to offset negative ones. Conversely, PBT posits that risk behaviors are not isolated acts but aggregate into a syndrome or cluster. Within this framework, one unhealthy behavior (e.g., smoking) increases the likelihood of adopting other risky behaviors, such as a poor diet or low PA, due to shared underlying factors. These theories provide complementary frameworks for interpreting adolescents’ complex behavioral profiles, one emphasizing compensatory mechanisms, the other a clustering pattern of co-occurring risks [10]. Moreover, longitudinal evidence indicates that “healthier” profiles are linked to lower adiposity and that clusters track over time, reinforcing the importance of acting early [8,11].

Consistent with previous studies [18,19], urban adolescents had higher odds of belonging to Class 1 and Class 2. This pattern may reflect greater access to FF and screens/technology and fewer opportunities or spaces for PA in urban settings [20,21]. Boys were more likely to belong to the multi-risk pattern (Class 3), echoing reports that boys engage more in certain unhealthy behaviors than girls [21], potentially due to differences in food preferences, leisure activities, and attitudes toward PA [20,21]. These patterns are hypothesis-generating and should be tested in future studies. In Morocco, contextual factors, including dietary norms, urban food environments, variability in PA infrastructure and safe public spaces, and ongoing nutrition/CSD policy efforts, may also shape these behavioral profiles.

Indicators of poor mental health (anxiety and suicidal ideation/plan/attempt) were associated with Class 2 and/or Class 3, consistent with prior evidence linking unhealthy behavior patterns and mental health problems [7,22]. Anxiety may contribute to impulsive food choices and avoidance of PA, potentially reinforcing a negative cycle [23]. Likewise, smoking co-occurred with the more adverse profiles, in line with studies showing clustering between tobacco use, poor diet, and insufficient PA [7,9].

This study is, to our knowledge, the first to examine behavior clustering among adolescents in Morocco, where such research remains scarce [14]. Strengths include a large, school-based sample drawn from a national survey and a person-centered LCA approach capturing co-occurrence rather than single behaviors.

At the same time, several limitations merit attention. First, analyses were unweighted, and the complex survey design (weights/strata/clusters) was not modeled. This is a major limitation that constrains national generalizability; point estimates are sample-specific, variance may be misestimated, and national generalizability is limited, warranting cautious interpretation. Second, the cross-sectional design precludes causal inference. Third, model entropy was moderate; class membership was assigned via modal classification (classification error not propagated), which may bias class-covariate estimates toward the null. No additional sensitivity analyses were undertaken to model classification error; however, any misclassification from moderate entropy is expected to attenuate associations rather than generate spurious findings. Finally, indicators were self-reported and dichotomized (entailing information loss) and may be affected by recall and social desirability bias; sleep and other behaviors were unavailable. Growing evidence suggests that sleep interacts with PA, SB, and diet within a 24-hour time-use framework; time spent in one necessarily displaces the others. Shorter or irregular sleep is associated with lower PA, greater sedentary/screen time, and less healthy dietary patterns [8,11].

Future work should therefore incorporate sleep (e.g., duration/regularity) as an additional indicator or covariate to test whether behavioral profiles shift when sleep is considered. Policy and practice implications include multi-component school-based programs (nutrition, PA, and media literacy), strengthened CSD policy implementation and school food standards, integrated brief mental health supports, and pilots to enhance active-transport and PA infrastructure. Research priorities include survey-weighted/three-step LCA, adding sleep and objective PA, longitudinal tracking, and multilevel school/neighborhood analyses.

## Conclusions

This study aimed to identify latent behavioral classes of UD, PA, and SB among Moroccan adolescents using LCA and to examine their sociodemographic and mental health correlates. A three-class solution fit best: low FV intake and inactive, CSD and FF intake and inactive, and multi-risk diet and inactive. Urban residence, male sex (for the multi-risk class), high-school level, anxiety, suicidality, and smoking were associated with higher odds of membership in the riskier classes, while underweight was linked to lower odds for the multi-risk class.

These findings support school-based, multi-component programs targeting diet quality, daily PA, and SB reduction, with brief mental health support and prioritizing boys, urban schools, and smokers. However, conclusions are tempered by unweighted, cross-sectional, school-based data; estimates are sample-specific and may not generalize nationally. Future work should use survey-weighted/three-step LCA and longitudinal, multilevel designs with objective measures and sleep to strengthen generalizability and causal inference.

## Appendices

**Table 5:** Covariates: definitions and coding *: Codes were developed based on the GSHS Data User's Guide and Morocco Public Use Codebook [6]. Detailed information on these resources can be accessed at https://extranet.who.int/ncdsmicrodata/index.php/catalog/649 **: Single-item hunger frequency is commonly used in GSHS analyses as a proxy for adolescent SES; it reflects perceived material deprivation/food insecurity. ***: BMI was computed as kg/m² and converted to age- and sex-specific BMI-for-age z-scores using the WHO 2007 reference (5–19 years). Categories were defined as: underweight < −2 SD; normal −2 to +1 SD; overweight > +1 SD; obesity > +2 SD.

Covariates	Item in the questionnaire	Options and coding*
Area	Self-reported by the participants	Coded 1 = Urban, and 0 = Rural
Sex		Coded 1= boys, and 0 = girls
Age (years)		Coded 1 = 13 years or less, 2 = 14-15 years, and 0 = 16 years or more
Academic level		Coded 1= High school, and 0= middle school
Socioeconomic status (SES)**	During the past 30 days, how often did you go hungry because there was not enough food at home?	1 = never to 5 = always (coded 1 = Low SES most of the time/always; and 0 = Medium or high SES never/rarely/sometimes)
BMI category (WHO BMI for age)***	- Height: How tall are you without your shoes on? (meters) - Weight): How much do you weigh without your shoes on? (kilograms)	Coded 1 = Underweight, 2 = Overweight or obese, and 0 = Normal weight
Loneliness	During the past 12 months, how often have you felt lonely?	1 = never to 5 = always (coded 1 = mostly/always and 0 = never/rarely/sometimes)
Anxiety	During the past 12 months, how often have you been so worried about something that you could not sleep at night?	1 = never to 5 = always (coded 1 = mostly/always and 0 = never/rarely/sometimes)
Suicidal ideation	During the past 12 months, did you ever seriously consider attempting suicide?	1 = Yes to 2 = No (coded 1 = Yes and 0 = No)
Suicidal plans	During the past 12 months, did you make a plan about how you would attempt suicide?	1 = Yes to 2 = No No (coded 1 = Yes and 0 = No)
Suicidal attempts	During the past 12 months, how many times did you actually attempt suicide?	1 = Yes to 2 = No No (coded 1 = Yes and 0 = No)
Cigarette smoking	During the past 30 days, on how many days did you smoke cigarettes?	1 = 0 days to 7 = All 30 days (coded 1 = 1 day or more and 0 = 0 day)

## References

[1] Patton GC, Sawyer SM, Santelli JS (2016). Our future: a Lancet commission on adolescent health and wellbeing. Lancet.

[2] Santarossa S, Sitarik AR, Johnson CC (2021). Associations of physical activity with gut microbiota in pre-adolescent children. Phys Act Nutr.

[3] van Sluijs EM, Ekelund U, Crochemore-Silva I (2021). Physical activity behaviours in adolescence: current evidence and opportunities for intervention. Lancet.

[4] (2018). U.S. Department of Health and Human Services. Physical Activity Guidelines Advisory Committee Scientific Report. Washington, DC: HHS; 2018. Accessed October 25. 2018 Physical Activity Guidelines Advisory Committee Scientific Report.

[5] (2025). U.S. Department of Agriculture; U.S. Department of Health and Human Services. Scientific Report of the 2015 Dietary Guidelines Advisory Committee: Advisory Report to the Secretary of Health and Human Services and the Secretary of Agroculture.

[6] (2025). World Health Organization: Global School-Based Student Health Survey 2016. https://extranet.who.int/ncdsmicrodata/index.php/catalog/649.

[7] Alosaimi N, Sherar LB, Griffiths P, Pearson N (2023). Clustering of diet, physical activity and sedentary behaviour and related physical and mental health outcomes: a systematic review. BMC Public Health.

[8] Blyth F, Haycraft E, Peral-Suarez A, Pearson N (2025). Tracking and changes in the clustering of physical activity, sedentary behavior, diet, and sleep across childhood and adolescence: a systematic review. Obes Rev.

[9] Leech RM, McNaughton SA, Timperio A (2014). The clustering of diet, physical activity and sedentary behavior in children and adolescents: a review. Int J Behav Nutr Phys Act.

[10] de Mello GT, Minatto G, Costa RM, Leech RM, Cao Y, Lee RE, Silva KS (2024). Clusters of 24-hour movement behavior and diet and their relationship with health indicators among youth: a systematic review. BMC Public Health.

[11] Alosaimi N, Sherar LB, Griffiths P, Hamer M, Pearson N (2024). Clusters of diet, physical activity, screen-time and sleep among adolescents and associations with 3-year change in indicators of adiposity. PLoS One.

[12] Matias TS, Alves JF, Nienov GT, Lopes MV, Vasconcellos DI (2023). Clustering of physical activity, sedentary behavior, and diet associated with social isolation among Brazilian adolescents. BMC Public Health.

[13] Weller BE, Bowen NK, Faubert SJ (2020). Latent class analysis: a guide to best practice. J Black Psychol.

[14] Mello GT, Lopes MV, Minatto G (2021). Clustering of physical activity, diet and sedentary behavior among youth from low-, middle-, and high-income countries: a scoping review. Int J Environ Res Public Health.

[15] Miranda VP, Coimbra DR, Bastos RR, Miranda Júnior MV, Amorim PR (2021). Use of latent class analysis as a method of assessing the physical activity level, sedentary behavior and nutritional habit in the adolescents' lifestyle: a scoping review. PLoS One.

[16] Van Kollenburg GH, Mulder J, Vermunt JK (2015). Assessing model fit in latent class analysis when asymptotics do not hold. Methodology.

[17] Li H, Zhang W, Yan J (2024). Physical activity and sedentary behavior among school-going adolescents in low- and middle-income countries: insights from the global school-based health survey. PeerJ.

[18] Kerkadi A, Al Mannai H, Saad D, Yakti FA, Attieh G, Bawadi H (2021). Clustering of lifestyle risk factors among Algerian adolescents: comparison between urban and rural areas: GSHS data. Int J Environ Res Public Health.

[19] Silva KS, Barbosa Filho VC, Del Duca GF, de Anselmo Peres MA, Mota J, Lopes Ada S, Nahas MV (2014). Gender differences in the clustering patterns of risk behaviours associated with non-communicable diseases in Brazilian adolescents. Prev Med.

[20] El-Ammari A, El Kazdouh H, Bouftini S, El Fakir S, El Achhab Y (2020). Social-ecological influences on unhealthy dietary behaviours among Moroccan adolescents: a mixed-methods study. Public Health Nutr.

[21] Abdelghaffar EA, Hicham EK, Siham B, Samira EF, Youness EA (2019). Perspectives of adolescents, parents, and teachers on barriers and facilitators of physical activity among school-age adolescents: a qualitative analysis. Environ Health Prev Med.

[22] Hoare E, Milton K, Foster C, Allender S (2016). The associations between sedentary behaviour and mental health among adolescents: a systematic review. Int J Behav Nutr Phys Act.

[23] Roohafza HR, Afshar H, Keshteli AH, Mohammadi N, Feizi A, Taslimi M, Adibi P (2014). What's the role of perceived social support and coping styles in depression and anxiety?. J Res Med Sci.

